# Enacting a social ecology: radically embodied intersubjectivity

**DOI:** 10.3389/fpsyg.2014.01321

**Published:** 2014-11-18

**Authors:** Marek McGann

**Affiliations:** Department of Psychology, Mary Immaculate College, University of LimerickLimerick, Ireland

**Keywords:** enactivism, ecological psychology, affordances, behavior settings, culture

## Abstract

Embodied approaches to cognitive science frequently describe the mind as “world-involving,” indicating complementary and interdependent relationships between an agent and its environment. The precise nature of the environment is frequently left ill-described, however, and provides a challenge for such approaches, particularly, it is noted here, for the enactive approach which emphasizes this complementarity in quite radical terms. This paper argues that enactivists should work to find common cause with a dynamic form of ecological psychology, a theoretical perspective that provides the most explicit theory of the psychological environment currently extant. In doing so, the intersubjective, cultural nature of the ecology of human psychology is explored, with the challenges this poses for both enactivist and ecological approaches outlined. The theory of behavior settings (Barker, 1968; Schoggen, 1989) is used to present a framework for resolving some of these challenges. Drawing these various strands together an outline of a radical embodied account of intersubjectivity and social activity is presented.

## IN SEARCH OF THE PSYCHOLOGICAL ENVIRONMENT

Many of the various flavors of embodied cognitive science describe the mind as “world involving.” Psychological activity is “situated” or “embedded”, dependent on or highly sensitive to environmental conditions. Enactive cognitive scientists quote the philosopher Merleau-Ponty to provide perhaps the most dramatic example of such thinking:

The world is inseparable from the subject, but from a subject which is nothing but a project of the world, and the subject is inseparable from the world, but from a world which the subject itself projects.

(Merleau-Ponty, 1962, p. 430)

Given such a view, understanding the mind requires an account of the psychological environment as detailed and comprehensive as our accounts of the cognitive system. I believe that enactivists have yet to provide such an account.

In order to address this issue, in this paper I will advocate for a closer alliance between enactive thinking and ecological psychology as it has developed from the work of James J. Gibson. In doing so I endorse a similar call by Chemero (2009), and explore some of the ways in which these two approaches can be brought closer together to the benefit of both.

Primarily, I will argue that drawing on the theoretical resources of ecological psychology offers significant benefits for an enactive cognitive science, though I will also note where I consider enactivism has something to offer ecological psychology. Further, following arguments that all of human psychology in particular is embedded not only in a physical but a social and cultural surround, I outline how a combined approach enables a comprehensive account of the human psychological environment.

In the following sections I will outline first the extant enactive thinking on the psychological environment and the core tenets of the related but distinct ecological perspective. I will then examine the revisions of traditional ecological thinking that Chemero (2009) uses to bring these two approaches into closer alignment and suggest some resolutions to remaining tensions. With this groundwork laid I turn to the question of sociality and the shared environment. Following the work of Heft (2007, 2011), I suggest that the concept of behavior settings advanced by Barker (1968) and others can be used to understand social activity and suggest this as an example of the kinds of theoretical resources that an ecological psychology can provide for enactive thinking. I argue that an understanding of behavior settings, encapsulated within a radical embodied framework, can form a sound basis for a science of embodied intersubjectivity.

## WHAT IS AN ENVIRONMENT BROUGHT FORTH BY ENACTION?

The enactive approach posits a fundamental complementarity between the agent and its environment. As the quotation from Merleau-Ponty makes clear, the two are seen as deeply interdependent. Enactivists describe agents and their environments as arising together, emergent phenomena (Varela et al., 1991; Weber and Varela, 2002; Thompson, 2007). For enactivists, it all begins with an autonomous, organisationally closed, system (see Varela, 1979). Such systems are made of a set of processes where each process depends on at least one other component and supports at least one other. Once such a system arises in the world the system operates so as to implicitly make a distinction between things (processes) that are part of that system and those that are not. The system, the most basic form of agency whose only purpose can be seen as continuing to produce itself (Weber and Varela, 2002; Thompson and Stapleton, 2009), will be structurally coupled to the world around it. Richer, more complex systems have richer, more complex potential interactions (Di Paolo, 2005; Barandiaran et al., 2009). Some aspects of the world are relevant to the agent’s concerns and body, and can affect it in various ways, whereas there are large portions of the world that are effectively absent or non-existent for the agent. The *environment*, then, is the world standing in various relations to the agent, relations that hold because of the agent’s values, needs, capabilities and embodiment. As a relational phenomenon the environment emerges with the agent, the two are a complementary pair and neither can be fully specified without reference to the other.

Thinking in such terms means that encounters between an agent and its environment are normally achievements of the agent rather than impositions upon it. The world does not stimulate a passive agent, but rather the agent engages with its surround; interaction is sought. Psychology is, by these lights, not a process of stimulus and response. There is no starting point for an organism’s actions (a trigger stimulus to a patient organism) because they are already alive, already acting, already concerned. Simply being alive means that an agent is coordinating its own activity with that of its environment. Enactivists term this process *sense-making*. An event, process, or object in the world only exists for the agent insofar as it affects and can be brought into coordination with the agent’s own on-going activity – it is the world made sense of by the organism. A classic illustration of this kind of coordination often used in the enactive literature is that of a bacterium’s climbing of a sucrose gradient (Varela, 1991).

The *Escherichia coli* bacterium has two modes of locomotion: one characterized by random tumbling, the other by coherent movement in a given direction. The chemical sucrose can interact with the bacterium’s cell membrane and can be metabolized by the cell. As such, an *E. coli* can encounter sucrose, and what is more, tends to encounter it as food. When a tumbling bacterium encounters sucrose it tends to switch to a more coherent movement that brings it toward areas of higher concentration of food. This illustration outlines the mutual character of the agent and its environment – the sucrose can only be present for the organism because the organism’s embodiment enables it. The agent simply cannot engage with many other aspects of the world (e.g., tectonic movements, most variations in the electromagnetic spectrum, most variations in atmospheric pressure). The example also makes the point that engagements between an agent and the environment involve the coordination of the agent’s needs or values (in this case the need of continued material self-production to which sucrose can contribute, serving the value of continued existence) with the resources, opportunities, threats, and demands of an environment that matters to it.

The enactive description of psychology fits very closely with the notions of Dewey (1896) set forth in his classic paper “The reflex arc concept in Psychology.” Dewey argues that a “response” is never “triggered” by a “stimulus” because the stimulus is always encountered in the process of the agent’s on-going behavior. Rather than consider stimuli and responses we are better considering tensions that arise in the organism’s encounters that are resolved by coordinations. Psychology is not a process that occurs in the space between stimulus and response but in the engagement between an agent and its environment. It is a relational phenomenon that must be addressed in relational terms that acknowledge both aspects of the tensions and coordinations in question.

Many of the illustrations of the world-involving nature of cognitive activity by enactive researchers deal in rather fundamental biological terms, such as the chemical processes in living cells (Varela, 1991) or minimalist computational robotics models that illustrate proofs of concept (Di Paolo, 2003; Di Paolo et al., 2010; Egbert and Barandiaran, 2014; Egbert and Cañamero, 2014). The characterisation of the relationship between the agent and the world in stark physical, chemical, or dynamical terms of bodily processes coupled to environmental ones makes some important points. The environment does not stand outside of the agent, imposing stimuli upon it in tit-for-tat exchanges of trigger and movement. It remains something of an open task for enactivists, however, to characterize the psychological environment in terms that fit both the enactive attitude – acknowledging the relational, co-determined nature of the environment and psychological activity – as well as experience and activities more personally familiar to us human beings.

## THE ECOLOGICAL PERSPECTIVE

Perhaps the most clearly and systematically developed account of the psychological environment available is that of the ecological psychology that traces back to the perceptual psychologist Gibson (1966, 1986). Much like the enactivists who would come later, Gibson described a complementarity between the organism and its environment. He notes that the organism’s environment is not defined by the kinds of purely objective measures of Physics, but rather in terms relative to the agent – ecological terms. When being introduced to someone you do not stand, say, 80 cm from them, you stand within arm’s reach to shake their hand. The psychological environment, then, should be described relative to the psychological agent who is engaged with it.

On first blush it might seem that this way of thinking could lead us very quickly into an unwanted solipsism, with each organism living in its own distinct environment. Gibson (1986, p. 43) resolves this concern with a single clear and seemingly obvious point. Perceivers move. While no observers can occupy precisely the same point of view at the same time, the environment they share can be moved around and explored. The same perspective can be taken by different observers at different times. The environment remains to be explored by all of the observers that share it over the duration of its existence. An environment is shared inasmuch as two agents can perceive and act on it in a similar manner, something that will be the case for almost all animals of the same species and indeed many animals of different species.

Understanding the psychological environment as described by ecological psychology, then, involves understanding the relationship between an animal and its ecological niche – those aspects of the physical world that are relevant to the animal’s needs and capabilities and within which the animal will spend its life. This relationship between need, capability and the world around the organism brings out perhaps the most famous of concepts that Gibson put forward – affordances.

The affordances of the environment are what it offers the animal, what it provides or furnishes, either for good or ill...It implies the complementarity of the animal and the environment.

(Gibson, 1986, p. 127)

For us humans, for instance, flat ground generally affords walking on, while a cup affords grasping. The surface of water affords walking on by a pond-skater but not for us. Affordances are opportunities, allowing an animal to fit their actions to the world around them, or obstacles, demanding effective actions to be overcome.

Within the ecological literature affordances are commonly seen as properties of the environment. While they might be animal relative (such as the affordance of a pond surface for walking) they remain proper to the environment. Reed (1996a) takes quite a strong stance on this position, holding affordances as being properties of the world ready to be engaged with by any animal and which can impose selection pressures on species over evolutionary timescales. A more standard mode of thinking on the issue sees affordances as dispositional properties, properties of the world that can be instantiated just in those instances where an animal with the appropriate capacity interacts with it. This perspective is particularly associated with Michael Turvey, Robert Shaw and William Mace (Turvey et al., 1981; see also Turvey, 1992).

From an ecological perspective perception is generally perception of affordances. We perceive our environment in terms of what it affords. Crucially this perception is direct – it needs no representations, computations or other “mental gymnastics” (Chemero, 2009). Direct perception is to a large extent a matter of successful coordination of our behavior with some relevant variable in the environment. Rather than the creation of a perceptual image, the activation of some encoded memory or the production of a mental model, perception is the ability to engage with the environment.

Acting and perceiving take place in a medium. For us land-living types that medium is generally the air, which is transparent and diffuse so as to allow light, sound and solid objects to move through it readily. In the case of vision, light, which typically suffuses the entire domain in which we are behaving, will move (reflect, bounce around) in a reliable manner that is given structure by the shape and texture of objects in the vicinity. By moving our eyes we can use the structure of the light to coordinate our movements with the objects, surfaces, and other things in our environment. The world is perceived directly via these structures in the ambient array of energy (light or sound, for instance) and chemistry (in the case of smells) rather than interpreted through the construction of representations or models.

This structure in light (or sound or other energy and chemical arrays around us) Gibson referred to as “ecological information.” Quite different to how information is commonly discussed in Cognitive Science, it is structure in ambient energy that is formed due to the structure of the environment.

A classic example of ecological information is how the dynamics of optical flow specify and thereby allow us to perceive, time to impact as we move toward something (Lee, 1974). As we approach an object elements of its visual texture tend to spread apart in our visual field. The rate at which this happens has a direct relationship with how long we have until we hit the object in question (if it’s in the middle of our visual field and stays there). This information is present not in the form of some encoding but in the relationship between movement and structure in the ambient light. It is a relatively simple affair (which is to say that it requires no “mental gymnastics” nor a cognitive system capable of same) for an animal to guide various movement-based behaviors according to this easily sampled relationship. No representation of the actual time to impact is required because that can be perceived directly via the optic-flow variable in question.

Ecological information is not “taken in” or processed with some model (however, sparse or rich) of the environment. Rather, an animal is able to attune to it, to use it as a means of coordinating its behavior with the environment. The psychological environment is the set of affordances that exists for the agent. Ecological information is the means by which an animal perceives those affordances. Perceiving occurs not as a passive reception of stimuli but as an active part of perception-action cycles, coordinations between the agent and its environment.

Gibson thus shares with enactivists (and, indeed, Dewey) the notion that in perception the agent is already acting. Actions are coordinations with the environment, not responses to it. While the enactive and the ecological clearly have much in common, however, there are a few considerations that stall any straightforward adoption by enactivists of an ecological account of the environment.

## A DYNAMIC RECONCILIATION OF ENACTIVE AND ECOLOGICAL ACCOUNTS, CHEMERO’S “RADICAL EMBODIED COGNITIVE SCIENCE”

In *The Embodied Mind*, Varela et al. (1991, pp. 203–204) explicitly oppose their enactive view to Gibson’s ecological one. They take issue with a seemingly fixed conception of affordances put forward by Gibson, arguing that such an approach does not adequately acknowledge the dynamic interdependence between the agent and its environment. They quote Gibson (1972, p. 239) as understanding affordances, and the ecological information that specifies them, as “there to be discovered.”

The ontological priority here of not just the world but the environment (the perspectival, relational, description of the world) is a form of philosophical realism that runs counter to the emergentist views of enactivists. A more observer-relative description of affordances put forward by Turvey, Shaw, Reed and Mace (Turvey et al., 1981; see also Turvey, 1992) is somewhat less objectionable (Varela et al., 1991). However, the idea that the world for the agent is exhaustively specified at any given moment by ecological information, thus leaving much of the texture and detail of the agent unnecessary in a description of a given engagement, remains counter to an enactive stance. Similarly, a proper explanation of the perception of (visual) affordances will require more than just an account of optics, however, ecologically specified (Varela et al., 1991).

Over several decades of ecological research, however, there has been a long-standing debate as to just how affordances should best be conceived and how their relationship with the agent should be understood (Heft, 1989; Turvey, 1992; Chemero, 2003, 2009; Jones, 2003; Michaels, 2003; Stoffregen, 2003; Withagen and Chemero, 2012; Withagen et al., 2012).

Recently, Chemero (2009), in refining our understanding of affordances, has explicitly sought to reconcile the ecological and enactive viewpoints under a banner of “radical embodied cognitive science.” In order to do this, Chemero has argued a number of points.

Firstly, he redoubles the emphasis on dynamic interaction with the environment that is part and parcel of an ecological approach. Chemero notes that while ecological psychologists have adopted dynamical thinking and the methods of dynamical systems science in a deep and thorough-going manner over the past decades, the orthodox conception of affordances (that associated with Turvey et al., 1981) does not show quite the same dynamic sensibility.

Now, affordances have always been dynamical concepts. A flying ball might afford catching, but only while in flight. A stationary or slow-moving cup affords grasping, but not one moving too quickly. But many affordances are sufficiently stable such that they are often discussed simply as properties of the object in question – the flat rigid surface of the ground affords walking on, for instance. However, even something so basic as the rigidity required for walking on need only remain long enough for me to perform the action in question. Non-Newtonian dilatant fluids, for example, such as a suspension of starch in water, can afford walking despite the rigidity only lasting as long as the impact of a person’s foot with its surface (Custard, 2014).

Affordances are dynamic things whose presence describes an opportunity for effective action, a possibility of coordination. In being such they say as much about the agent acting as they do the environment with which they are engaged. Chemero (2009, p. 140) follows Michaels (2000), who argued that an affordance to punch a falling ball is perceived as “it’s time to flex the elbow.” By this view, affordances are not properties of the environment. They are, rather, relations that hold between an agent and their environment. In making this claim Chemero removes a significant point of disagreement between ecological and enactive thinking and asserts a relational description of the psychological environment. Chemero is still a realist about affordances, because affordances really do exist, but it is, as he put it “not a simple form of realism” (Chemero, 2009, p. 150). It is a realism that seems quite consistent with the emergentist commitments of enactivism.

Chemero also addresses considerations about how an organism might perceive affordances. The orthodox Turvey et al. (1981) view on the matter requires a strict one-to-one relationship between the ecological information (e.g., structure in the ambient array of light) and the affordance that it specifies. These must be lawfully related (even if the laws in question are specific to an ecologicalniche). Chemero (2009) uses the situational semantics of Barwise and Perry (1981) to dilute the lawfulness requirement. Like the philosopher Millikan (2000), he argues that the relationship need not be exception-free, it just needs to be sufficiently reliable to guide behavior effectively under normal circumstances, and, we might imagine, within normally recoverable bounds of likely perturbation or failure. This move offers some flexibility in the relationship between the agent and their environment that undermines the kind of objectivist pre-specification of relationships that Varela et al. (1991) considered counter to the enactivist emphasis on the role of the specific embodied agent with its own history of coupling with the world.

This perspective, more sensitive to individual histories and dynamics, is also present in Chemero’s view as he argues for another dynamic aspect to affordances – the gradual transformation of affordance relations over various timescales. Traditional thinking on affordances links them to the organism’s ecological niche, noting that over evolutionary time aspects of the environment become available for use by members of a species. Chemero points out that this also happens at a personal level over developmental time. It involves, in a sense, the construction of an individual, personal eco-niche, as a person develops certain skills or abilities and learns to engage with their environment in different ways.

Chemero (2009) refers to this niche for the individual organism as the phenomenological-cognitive-behaivoral niche of the particular animal. It is a concept intended to enable a more fine-grained analysis of the animal environment system. Rather than examining the effect of populations of animals on their shared environment, the focus is on the peculiarities of a single agent’s effect on the world around it. This will include the agent’s continually increasing sensitivity to the specific, particular details of that world, that give rise to a unique perspective. Such phenomenological-cognitive-behavioral niches will certainly be largely shared between animals with similar capacities, but will differ insofar as the particular histories and capabilities of those animals differ.

This fitting of the agent with its environment over time is achieved through another relation that holds between the two, one that is complementary to the affordance relation. Chemero (2009) describes *abilities* as relations that mediate changes in the animal (Chemero emphasizes the nervous system, but there is no *a priori* reason to limit the scope to that) that enable the organism to become sensitive to affordances.

In his outlining of the notion of abilities and the timescale over which they change Chemero focuses primarily on developmental time, the kinds of periods over which we learn new skills and gradually change what we are capable of. These changes tend to occur over much shorter timescales too, though. Central to enactive theorizing is the notion that the agent-enviornment system is valenced, normative. Enactivists, to a much greater degree than mainstream ecological thinking, emphasize the importance of motivation and intentions. In addition to more stable species-typical capabilities and even individually tuned skills, the immediate field of action of an agent will depend on the flow of it needs and intends at a given time.

### THE ENGAGEMENT: THE FIELD OF ACTION OF INTENTIONAL AGENTS

In a brief paper that provides an overview of enactivist psychology, McGann et al. (2013) claim that enaction begins with an engagement – a particular encounter between an agent and their environment. For enactivists psychology is to be found in the entire animal-environment system, and ecological psychologists hold to the same idea. Both points of view find an agent already dealing with being alive, already interacting with its environment, rather than waiting in passivity and darkness for stimulation. The ecological perspective (and Chemero’s revision of it still shares this characteristic) examines how the process of the interaction unfolds or develops over time, the dynamics of the sensorimotor processes. Enactivists have a similar interest, but also explore the various ways in which the biological dynamics of the living agent motivate or drive (and constrain) those sensorimotor processes (McGann, 2007; Di Paolo et al., 2010; Barandiaran and Egbert, 2013; McGann et al., 2013). Though substantial work remains to be done on this issue, particularly in “scaling up” to the complexities of human psychology, enactive theory makes salient considerations of intentionality (that is the formation and dynamics of intentions to act) about which ecological theorists have had comparatively little to say.

A notable exception on this front is the intentional theory of affordances advanced by Heft (1989). In an account that I believe is largely consistent with Chemero’s, Heft argues that in order to understand affordances we must not only describe them relative to the body of the agent, but to that body in the process of intentional activity. This provides a much more dynamic and relational conception of affordances than the ecological psychology orthodoxy. As motivations wax and wan the relevance of different abilities varies and the engagement between agent and environment varies accordingly. Heft (1989) notes that intentions must themselves be considered in world-involving, relational, terms – these are not mentalistic representations after all – and to leave them out of the description of the agent’s relations with its surround is a mistake.

Chemero’s description of abilities has no prominent role for these intentional, motivational aspects of the agent’s activity, but no description of an engagement, an animal-environment system, can be complete without them. Along with the driven, valued, normative character of the engagement, they also highlight the short-timescale dynamics of abilities and affordances, which will arise and dissolve as relations as the agent finds its values challenged or facilitated, in conflict or coordination, in interaction with its environment. We might describe the general ecological niche of a given species, and even a particular animal’s phenomenological-cognitive-behavioral niche, but animals don’t interact with generalities. These broader descriptions of an environment provide progressively higher resolution explanations for an animal’s behavior. Understanding the finer-grained details of an organism’s activity on a given occasion will need to include the kind of fast-moving intentional dynamics that are involved in the engagement in question.

The engagement, the field of action of an agent, is defined by a complement of ability/affordance relations, with the proviso that these relations have a normative, intentional aspect. These relations have value. Sense-making was described above as the process of an organism being sensitive to and integrating the world into its own activity (at the very base, the activity of continually producing itself, staying alive). Insofar as something in the world plays a role (is an opportunity or threat of some kind) in the agent’s normative activity, the agent can make sense of it through the coordination of its behavior with the event, process, or object in question. Motivations and intentions are how we describe these normative aspects of an agent-environment system, and so sense-making is effectively a process of the coordination of an agent’s values and intentions with its environment.

Of course things get a little more tricky when there’s more than one agent in that environment.

## SHARED FIELDS OF ACTION

Where more than one agent is involved in a situation then the engagement is not just the coordination of one set of values or intentions with the environment, but a set of complex interactions between the various agents and their shared environment. Where the meaning or sense-making in the individual case is in the congruence between abilities and affordances that hold between agent and environment, in the social case there will be a set of relations that are negotiated between the agents. Whether another agent is an obstacle or resource, impediment, or aid to a given agent’s intentions is often malleable, due to the adaptive responsiveness of both agents to each other.

The variability of agentive action is in theory a significant challenge for an ecological approach to understanding the environment. The range and variability of animals’ behavior could be thought to undermine the reliable relationship between structure of ambient energy at any time and the animal’s activity. During any given period it is conceivable that the same person might engage in any one of numerous possible behaviors, some of which will share postures, gestures, or other physical attributes that give rise to structure in, for example, ambient light. Social interaction seems to our intuitions to be so pregnant with possibility that effective interpersonal engagement cannot be accounted for by the kind of direct, ecological mode of description I have been advocating here. Even allowing for Chemero’s somewhat less stringent relationship between environmental structure and perceived event there seems to be a want of reliability when dealing with other people, given just how diverse a single individual’s repertoire of behavior can be.

Of course this is a straw man of variability, because human behavior is rarely if ever that arbitrary or unpredictable. The question arises though, as to what provides the stability that human activity tends to have and how it channelises behavior such that the logically conceivable problem rarely ever arises in practice.

Heft (2007) has argued that a completely realized ecological psychology will in fact be social to its core. He claims that social activity is a fundamental part of the fabric of human psychology and must be a fundamental part of a complete ecological psychology. Drawing on paleoanthropology he notes that sociality is not just part of our evolutionary heritage, but part of our evolutionary history. *Homo sapiens* evolved in culture, not the other way around (Heft, 2007; see also Donald, 1991, 2001a,b and Tomasello, 1999a for related arguments). The mutual influence between animal and environment over time is a central tenet of ecological psychology – the organism’s ecological niche makes demands of and shapes the behavior of the organism, and in turn the organism over time affects the niche. Throughout the process of development, then, our behavior forms within and is shaped by our culture. Two facets of this process can be quickly identified.

The first facet is the process of behavior shaping that Merlin Donald has termed “deep enculturation” (Donald, 2001a). The idea is that during development a complex of standard ways of doing things is formed through which more intricate coordinations with our native culture are enabled. The ecological psychologist Reed (1993) put forward a distinct but related idea in what he terms the “field of promoted action.” Societies tend to evoke some behaviors more than others and in doing so shape the habits and capabilities of their members over the course of development. This of course has the effect of stabilizing behaviors, constraining the innumerable (or at least very numerous) possible activities in which a person might engage within some reliable range.

One of the principle means by which the field of promoted action is produced is the careful design and structuring of the physical environment (Reed, 1996b). This cultivation and curation of the environment in which we behave is the second facet of development that makes social interaction more reliable.

Gibson (1986) discusses the notion of *places*. Places are areas of the environment with a set of functional properties – they enable affordances for various specific activities. Over evolutionary, historical, and developmental time the physical environment has been nurtured to given ends, and distinctions between places sharpened. Much of our social activity, our shared and inter-coordinated behavior, is conducted in physical environments that support it. Examining this interdependence of the social and physical in some depth, Heft (2001, 2007, 2011) has shone a particular spotlight on the theory of behavior settings developed by Barker (1968) and Schoggen (1989). Developed independently of Gibson’s work, Heft has nevertheless argued that the theory of behavior settings is a effectively a theory of Gibson’s “places.”

### BEHAVIOR SETTINGS AS A THEORY OF PLACES

A behavior setting involves a cohesive set of standing patterns of behavior and those patterns’ physical surroundings (Barker, 1968). Easily overlooked and underestimated because of their near omnipresence in our lives we can nevertheless recognize examples of setting kinds immediately – a soccer game, a mathematics lesson, a religious service, a conference talk. They involve a set of physical resources, which often provide a spatial boundary to the setting (e.g., the walls of a classroom or church) as well as structuring the behavior of those within (perhaps with so blunt a means as a rigid arrangement of furniture). They also tend to have quite clear temporal boundaries. Specific instances of a behavior setting will form, evolve and dissolve at given times, often explicitly stated (e.g., a Wednesday, 10.30–11.10 mathematics lesson in classroom B6). Probably a majority of our lives is spent in different behavior settings (Heft, 2007).

In Barker’s (1968) original work examining the natural flow of behavior of residents in a small town, he and his field team found that the differences between the behavior of individuals tended to be greater within a person between settings than between people within settings. They also found that settings were just as powerful, if not more so, than identified antecedent stimuli in predicting the behavior of a person in their natural environment.

The theory of behavior settings is a rich and detailed one, whose apparent power unfortunately seems matched by its obscurity (Scott, 2005). For our present purposes it serves as a means for illustrating how cultural practices are enmeshed with physical surroundings and how the stability of physical environments is used to help stabilize social interactions.

With behavior settings in mind we can conceptualize deep enculturation as a process of learning how to engage with and make use of resources in our environment that are shaped and made available by a history of cultural practice. Enculturation is the cultivation of abilities to use socially provided and promoted resources, opportunities for shared and sanctioned actions.

Heft (2001) argues that the physical settings (Barker uses the unfortunate term “synomorphs”) which are complementary or similar in structure to the behaviors they support (they are “synomorphic” to the behavior) can be considered affordances for joint action. Many of the places in which we spend our lives are selected and designed to support the coordination of multiple people in some activity. More, the character of the physical environment and the inertia of encultured habits can lead settings to coerce the behavior of their inhabitants. Heft (2001) puts it as follows:

The relation between milieu and behavior is not contingent. It is not the case that because this room worked well as a classroom on previous occasions that it can be used for that purpose again. Rather it worked well on previous occasions (or not) because of its structure or form.

Because the meaning of the setting resides in the congruence between behavior and milieu, this relational structure has the potential to bring actions of individuals entering the setting in line with its functional character.

(Heft, 2001, p. 288)

Enculturation, through the promotion of certain patterns of behavior, substantially reduces the kind of variability in behavior that might be conceived as challenging a radical embodied (enactive, ecological) account of social interaction. Our subjectivity is at any time constrained by our shared environment, shared histories and shared abilities or habits.

This capacity for cultural background and social activity to constrain and shape our behavior brings into focus a final complication, a quirk of social dynamics that has seen some significant discussion over the past few years within enactivist thinking: the autonomy of the social.

### SITUATED PARTICIPATION: BEING DEEPLY ENGAGED WITH OTHERS

De Jaegher and Di Paolo (2007) noted that there are occasions when a social interaction can be more than the sum of its parts – situations in which the interaction takes on something of a life of its own. These situations, in which the participants together find themselves coordinating with each other perhaps despite their individual intentions, or coordinating with their environment in a manner not possible for either individually, are examples of “participatory sense-making.”

An important aspect of participatory sense-making is that the social dynamic is emergent. The social interaction is not merely a combination or aggregate of the behavior of its participants but is autonomous, it has a dynamic of its own that can constrain the behavior of the interactants just as much as facilitate it. The autonomous organization of the social dynamic provides it an inertia, making the interaction resistant to perturbation, perhaps even by the individuals enacting it. Whenever we have found ourselves in a conversation we couldn’t get out of (when both participants want it to stop), or felt an interaction drawn on an unwanted trajectory despite the efforts of both parties to prevent it, we are experiencing the autonomy of that interaction. An example used by De Jaegher and Di Paolo (2007) is that of two people trying to pass one another in a narrow corridor and being briefly unable to do so because of the way their behavior becomes coordinated – a brief back-and-forth “dance.”

For our present purposes what we take away from the idea of participatory sense-making is an admonition that engagement with a social situation is constrained not only by the ability/affordance relations of the participants but also by the inherent dynamics of the interaction itself. This over-riding dynamic, whether due to our culturally inherited resources, the inertia of habitual practice or our tendency to synchronize the rhythms of our actions with the environment (and the behaviors of others), can impose tensions and create perturbations in an agent’s activities as much as they might enable or facilitate them (De Jaegher and Froese, 2009). In situations of participatory sense-making we will need to describe the shared engagement in terms that are more than the aggregate of the individual engagements that comprise them.

Participatory sense-making as it is currently theorized is an important phenomenon that occurs in some but not all social interactions. If the actions of individuals are explained by the evolution of the agent-environment systems in question, the arising of tensions and coordinations between the two, there will be some circumstances in which the explanation of the actions of two or more interacting agents might produce a remainder – where their actions were in fact more than the sum of their parts, where the group of agents together were a single entity engaged with their environment rather than an aggregate of individuals. Behavior settings and the notion of places remind us that participatory sense-making will not occur in a vacuum but often in a cultivated physical milieu. These concepts offer a first pass theoretical account of how such over-arching dynamics can arise and can have functional effects. Barker (1968) and his colleagues have explored some of the ways in which settings coerce behavior, examining optimally- and under-inhabited settings and the different ways in which people respond to the requirements of a given place. These have also been put to some practical use in, for example, promoting inclusiveness in school-aged children (Fuhrer, 1993). A sensitivity to the broader context of a given activity offers some possible value in predicting when participatory sense-making is likely to occur, and what the course of its dynamic over time is likely to be.

Participatory sense-making reminds us that social activity is not just *more* activity, but is different in kind from interaction with the inanimate environment. However, the ideas of behavior settings and the acknowledgment of the socially curated, designed nature of most of the places in which human activity takes place equally remind us that participatory sense-making and the other complexities of social interaction are both supported and constrained by a host of observable and investigable factors. Recent work by Froese et al. (2014) is an example of how the dynamics of social interaction have been examined explicitly in these terms in a minimalist virtual environment. The theory of behavior settings offers a means of analyzing environments to explore the issue in more naturalistic contexts.

## RADICAL EMBODIED INTERSUBJECTIVITY

As has already been noted, a radical embodied approach that combines enactive and ecological thinking sees perception and action as occurring within an already flowing stream of activity. A living agent is never entirely at rest (even sleep is an activity). Such a view thus adopts a Deweyan notion of tensions and coordinations of behavior in context. When we are considering human beings the dynamics of tensions and coordinations are shaped by the practices and places of the surrounding culture.

Traditional, computational, or cognitivist models of psychology begin with a bare, decontextualized psychological system and layer context in the form of interpretations or biased representations over what are imagined as at least potentially faithful encodings of an external environment. For the view advanced here perceiving is done within the flow of behavior and so objects or actions of others show up in that flow, are engaged with as concordant or discordant with it. Interpretation doesn’t come after the fact, culturally formed cognitive activity is not an add-on or appendix to normal cognitive activity. Because in the human case abilities, habits, and practices are cultivated according to cultural norms from our earliest experiences, our culture does not introduce bias or add skew to our behavior, but inheres in the very basic forms of our activity from the get-go. Our acting and perceiving is done in cultural settings – in places – and our abilities (and their complementary affordances) develop accordingly.

Tomasello (1999a,b) has argued a similar point. He criticizes Gibsonian researchers for overlooking the cultural context in which objects are first encountered and the manner in which this affects people’s sensitivity to those objects’ affordances. He suggests the idea of “intentional affordances,” which are the normal functions to which objects are put and will be primary for that object in the field of promoted action. Here, I point out that this mode of thought generalizes to the social activity itself.

Just as perceiving and acting occurs within an on-going flow of activity, so people and their behavior are always present within a flow of cultural practice. We cannot identify and examine perceiving and acting separately to the context in which they show up, but must analyze them within the engagement between the agent and the environment. Similarly, we cannot pick out the individual cognitive processes or actions separately from their cultural context and attempt to understand the whole as the sum of its parts. A radical embodied approach requires us to always address phenomena as occurring as wholes, with parts existing insofar as they stand in various relations within that identified system. In the case of psychology, parts arise from wholes, rather than the other way around.

This approach imposes some challenges on us as investigators. A cognitive science must specify the context of its observations at all times, making explicit the situation in which the processes of interest are arising. While there may be ways in which aspects of context can be held steady across observations and even experiments, we can never leave implicit the particular dynamics of the setting in which behavior is emerging and flowing.

A radical embodied understanding of both individual and joint activity places that activity out in public – in the observable interaction. Intentions, actions, emotions and other phenomena are not locked away in the heads of participants, needing a series of inferences to identify them. We perceive these things directly insofar as we can coordinate effectively with them, whether that activity involves scientific observation or just personal interaction. To that extent, science is a direct extension of the personal activity of making sense of things (and in fact, is a contextualizing support for sense-making for those of us who are practicing scientists).

An understanding of intersubjectivity is approached from precisely the same perspective, seeing the individuals show up within the engagement rather than seeing the engagement as the linear sum of the actions and interpretations of rigidly specified individuals as they meet.

There is, thus, a sense in which you are a different person in different interactions, but the stability of your bodily dynamics and the inertia of your habitual behaviors, cultivated over time, within cultural contexts, means that you are not created anew, without history every time. The identity of individuals within interactions varies between situations but neither arbitrarily nor entirely unpredictably. Your role in a behavior setting will shape your behavior, as will your personal history of experience with such settings, and such roles. Many interactions will enable multiple social roles to be played and their associated skills exercised, other roles will be suppressed, or starved of opportunities.

Interacting with my undergraduate students, for instance, it is demanded of me that I play a didactic role and deploy a particular complex of skills in doing so. There is also occasional possibility for indulging in a little philosophical speculation but little if any possibility or likelihood in passing a soccer ball or debating the merits of a science fiction novel. A classroom setting can make certain demands on my because of my history and skillset – it makes different demands on my students.

Emergent interpersonal engagements are not fully autonomous from their enabling conditions – they still occur between embodied agents who are coupled to their environments (including each other) through various sensorimotor abilities. The utterance of a promise, a protestation of love or a glint in the eye still produce structure in the ambient energy of the living medium with which attuned agents can coordinate their actions. Though there is an important sense in which it is autonomous and the social domain has a dependence on recent history of the individuals’ interaction that the inanimate world does not – the same structure in the ambient array provides ecological information (supports effective coordination of action) under one history but not under another. What is more, because the relation is continually evolving, being negotiated, based on the actions of the agents involved some affordances for joint action will only arise when other aspects of behavior have been effectively entrained and the two are involved in participatory sense-making.

Attempting to reduce participatory sense-making to the actions of individual participants is doomed to failure, but the autonomy of social practice is still conducted by embodied agents in physical settings and these emergent dynamics can be explored by examination of these enabling and constraining features.

## DIRECT SOCIAL PERCEPTION

One of the concerns that critical readers might raise is whether direct perception is really possible in activity that is so heavily mediated by cultural processes. How can it be the case that I directly perceive, say, an insult, given that the host of cultural and historical dependencies on which such an experience is based? Surely there must be some representation that the cognitive system must use to keep track of relationships and enable the rich complexity of even momentary events in social interactions.

This kind of concern makes two mistakes.

First, direct perception is not a claim that what is perceived is unmediated. Cultural events and actions are mediated by tradition and practice, but those events can still be directly perceived. Cognitivist and computationalist models of psychology have perhaps trained our intuitions to consider that only the world as described by Physics, in its neutral, raw, brute form can be perceived directly. To perceive culturally mediated phenomena such as social roles, symbols, and the social implications of actions *must* require mental gymnastics to infer the cultural import of a physical event.

Direct perception of non-physical (in the “mere” or “brute” sense of physical) is a perfectly coherent notion and all of ecological psychology is grounded in the idea. For ecological psychology the pickup of ecological information is done through physical interaction, of course (what else could it be?) but what that information enables perception of can be anything so long as a sufficiently reliable relationship exists between it and the information in question. The glint in my wife’s eye or the rudeness in the exclusionary orientation of a person’s body, or of the offensiveness of their utterances, are perceived within the interaction, not built, LEGO-style, from the perception of their elements. They depend on my ability to engage effectively with social practices and in the individual people in question, but as I have noted those abilities are culturally shaped from the ground up. My movements and utterances are culturally structured, meaningful at their most basic level; cultural relevance and value is not added afterward.

Second, direct perception is not instantaneous (Bingham, 1995). It is un-mediated by inference or representations, but it can still take time, sometimes quite a long time. Because of the dynamic nature of the relationship at least some time (even if it’s a very very short period of time) will be required to allow the agent to coordinate their behavior. However, where the dynamics of the environment are slower, then the process of perceiving might take relatively prolonged periods. It can take time to see another person’s intentions and different periods of time might make different aspects of the other person perceivable. Over increasingly long durations we may see only the contours of the other’s intentions, then their general thrust and tone, and finally their finer grains. Direct perception can be slow, and what is perceived can be vague. There is also no particular moment in time at which perceiving is “complete” because such perception always occurs in the flow of on-going behavior – activity does not have to wait for it.

For more cognitivist thinkers any prolonged coordination will imply the existence of a representation capable of being updated so that the agent can keep track of details as they become apparent. This mode of thought, however, makes the assumption that at any given time the agent’s interactions with the environment are being built up from bare physical facts that need interpretation, and are overlooking the possibility of an on-going process of activity whose trajectory is amended as it is perturbed or otherwise constrained by the way in which it is coupled with the environment.

Historical dependency of processes is something that is inherent in a great many forms of dynamical system, with no need for representations to keep track of that history. Social relationships between agents are particularly sensitive to historical dependencies.

## SUMMARY AND CONCLUSION

Dewey (1896) argued that no behavior occurred outside of the context of the animal’s already on-going stream of activity. Perceiving and acting exist in a dynamic of tensions and coordinations that enable the continuity of a person’s effective coping in the world. The “parts” of psychological activity emerge out of the “whole” of a living being’s engagement with its environment, not the other way around.

Enactive and ecological approaches to cognitive science developed independently, but effectively extend and flesh out Dewey’s insight. In doing so, they highlight the need for a characterisation of both the embodied psychological agent, and the environment, in terms that acknowledge their interdependent relationship. I have argued in the present paper that bringing enactive and ecological points of view together offers the best hope for such an account, over either perspective alone [and in this I offer an initial response to a call for their closer alignment by Chemero (2009)].

The “already acting” point of view that this account involves means that the environment is never encountered ahistorically. All acting and perceiving is done in a flow of activity that is continuous for living beings. For us human beings the fields of action, the engagements in which we find ourselves, have both personal and cultural histories. Our subjectivity is dependent on our intersubjectivity. Social activity mediates individual psychology but does so in a manner that is fundamental, not additional. Cultural activity does not sit on top of more basic forms of behavior. Rather, it evokes, shapes and transforms those basic actions. The environment in which we human beings live and act is cultural to its core.

The approach advocated here poses some challenges for empirical investigation, but can also draw effectively on established theoretical resources, particularly in the form of the theory of behavior settings of Barker (1968) and Schoggen (1989). As we look to the horizon of a more culturally sensitive embodied cognitive science it might also be possible to begin a process of integration with some aspects of cultural psychology (Bruner, 1990; Harré, 1998; Benson, 2000; Harré and Moghaddam, 2012) where the primacy of cultural practice in psychological activity is already acknowledged.

By these lights, a science of radically embodied intersubjectivity is not only possible, it is the only way in which we can adequately address the question of the nature of the human mind.

## Conflict of Interest Statement

The Editor Hanne De Jaegher declares that, despite having collaborated with author Marek McGann, the review process was handled objectively and no conflict of interest exists. The author declares that the research was conducted in the absence of any commercial or financial relationships that could be construed as a potential conflict of interest.
